# Efficacy of navigation system-assisted distraction osteogenesis for hemifacial microsomia based on artificial intelligence for 3 to 18 years old: study protocol for a randomized controlled single-blind trial

**DOI:** 10.1186/s13063-023-07809-9

**Published:** 2024-01-12

**Authors:** Xiangqi Liu, Ziwei Zhang, Wenqing Han, Zhijie Zhao, Byeong Seop Kim, Yingjie Yan, Xiaojun Chen, Xuetong Wang, Xin Li, Xianxian Yang, Bingshun Wang, Haisong Xu, Li Lin, Gang Chai

**Affiliations:** 1 Department of Plastic and Reconstructive Surgery, Shanghai 9Th People’s Hospital, School of Medicine, Shanghai Jiao Tong University, 639 Zhi Zao Ju Rd., Shanghai, 200011 China; 2 Department of Biostatistics, Clinical Research Institute, Shanghai Jiao Tong University School of Medicine, 227 Chong Qing Nan Rd., Shanghai, 200025 China

**Keywords:** Electromagnetic navigation, Hemifacial macrosomia, Mandibular osteotomy

## Abstract

**Background:**

Mandibular distraction osteogenesis (MDO) is a major part of the treatment for hemifacial microsomia patients. Due to the narrow surgical field of the intraoral approach, osteotomy accuracy is highly dependent on the surgeons’ experience. Electromagnetic (EM) tracking systems can achieve satisfying accuracy to provide helpful real-time surgical navigation. Our research team developed an EM navigation system based on artificial intelligence, which has been justified in improving the accuracy of osteotomy in the MDO in animal experiments. This study aims to clarify the effect of the EM navigation system in improving the MDO accuracy for hemifacial microsomia patients.

**Methods:**

This study is designed as a single-centered and randomized controlled trial. Altogether, 22 hemifacial microsomia patients are randomly assigned to the experiment and control groups. All patients receive three-dimensional CT scans and preoperative surgical plans. The EM navigation system will be set up for those in the experiment group, and the control group will undergo traditional surgery. The primary outcome is the surgical precision by comparing the osteotomy position of pre- and postoperative CT scan images through the Geomagic Control software. The secondary outcomes include mandibular symmetry (occlusal plane deviation angle, mandibular ramus height, and body length), pain scale, and complications. Other indications, such as the adverse events of the system and the satisfactory score from patients and their families, will be recorded.

**Discussion:**

This small sample randomized controlled trial intends to explore the application of an EM navigation system in MDO for patients, which has been adopted in other surgeries such as orthognathic procedures. Because of the delicate structures of children and the narrow surgical view, accurate osteotomy and protection of nearby tissue from injury are essential for successful treatment. The EM navigation system based on artificial intelligence adopted in this trial is hypothesized to provide precise real-time navigation for surgeons and optimally improve patient outcomes, including function and aesthetic results. The results of this trial will extend the application of new navigation technology in pediatric plastic surgery.

**Trial registration:**

Chinese Clinical Trial Registry ChiCTR2200061565. Registered on 29 June 2022.

## Administrative information

**Table:** 

Title {1}	Efficacy of navigation system-assisted distraction osteogenesis for hemifacial microsomia based on artificial intelligence for 3 to 18 years old: study protocol for a randomized controlled single-blind trial
Trial registration {2a and 2b}	ChiCTR2200061565. Registered on June 29, 2022 (https://www.chictr.org.cn/showproj.aspx?proj=168053)
Protocol version {3}	December 16, 2021 Version 1.1
Funding {4}	Clinical Research Plan of SHDC [No. SHDC2020CR3070B]; the project of Shanghai Municipal Key Clinical Specialty [No. shslczdzk00901] and the project of the Clinical Research Program of 9th People’s Hospital affiliated to Shanghai Jiao Tong University School of Medicine [No. JYLJ202108]; Shanghai Jiao Tong University School of Medicine Two-hundred Talent [No.20161420]
Author details {5a}	^1^Department of Plastic and Reconstructive Surgery, Shanghai 9th People’s Hospital, School of Medicine, Shanghai Jiao Tong University, 639 Zhi Zao Ju Rd, Shanghai, 200,011, China ^2^ Department of Biostatistics, Clinical Research Institute, Shanghai Jiao Tong University School of Medicine, 227 Chong Qing Nan Rd, Shanghai 200,025, China
Name and contact information for the trial sponsor {5b}	Li Lin, Department of Plastic and Reconstructive Surgery, Shanghai 9th People’s Hospital, School of Medicine, Shanghai Jiao Tong University, 639 Zhi Zao Ju Road, Shanghai, 200,011, China Gang Chai, Department of Plastic and Reconstructive Surgery, Shanghai 9th People’s Hospital, School of Medicine, Shanghai Jiao Tong University, 639 Zhi Zao Ju Rd, Shanghai, 200,011, China
Role of sponsor {5c}	The funding agency did not participate in the study design, data collection, or any other part of the trial. The sponsor declares that there is no commercial interest in the trial

## Introduction

### Background and rationale {6a}

Hemifacial microsomia (HFM) is the second most common congenital dysplasia of the face after cleft lip and palate, with a prevalence of 1:3000–5000 live births [1]. The patients present asymmetric structures derived from first and second branchial arches, including hypoplastic mandible resulting in the deficit of muscles of mastication except for the masseter, deformed auricle/microtia/ anotia, and some patients even may have microphthalmia [1, 2]. It was reported that 49–100% of patients suffer mandibular hypoplasia, and patients may have mild hypoplasia or lack of temporomandibular joint (TMJ) [1]. The wildly adopted Pruzansky classification helps describe mandibular deformity [3].

Mandibular distraction osteogenesis (MDO) was popularized by McCarthy in 1992 to treat mandibular hypoplasia [2, 4]. In the MDO procedure, a single osteotomy occurs at the mandible ramus through the medial and lateral cortices in the space between the mandibular angle. The distraction forces are applied by a device fixed by screws on either side of the osteotomy line [4].

Planning the osteotomy plane is critical to avoid the injury of tooth roots, the inferior alveolar nerve, and the marginal mandibular branch of the facial nerve. It is also vital to the distraction vector for successful MDO and the final aesthetic result [5]. However, most of this procedure has been done intraoral. The narrow view and operative space of MDO in young patients increase the surgeons’ difficulty in achieving precise osteotomy. The development of versatile computer-assisted equipment and methods helps surgeons to locate the osteotomy during the procedure [4–7].

Our previous research adopted a computer-aided-designed (CAD) surgical guide to locate the osteotomy line during the surgery [5]. While the surgical guide has been designed preoperatively based on the patient’s CT scan and surgical simulation, the surgeons must put it on to the ramus in the narrow intraoral surgical incision, which is difficult, especially for very young children, and often needs to expand the incision which may cause extra injury. Therefore, navigation systems are invented as a great alternative to improve accuracy [7–10].

In recent years, scads of studies have reported that electromagnetic (EM) tracking systems can achieve satisfying accuracy. The EM sensors are usually small and delicate, allowing more free movement between the sensors and the generator [6]. Our team has developed a maxillofacial surgical robot system (MSRS) based on artificial intelligence surgical stimulation and EM navigation technology, which has been proven feasible for mandibular angle osteotomy in animal experiments in terms of accuracy and workflow of the procedure [11, 12]. Therefore, we proposed the MSRS system to clinical practice for hemifacial microsomia patients to explore the value of this system in improving the accuracy of osteotomy for our young patients.

## Objectives {7}

The primary objective of this trial is to assess the accuracy of electromagnetic (EM) tracking systems based on artificial intelligence in osteotomy for HFM patients, which is evaluated by measuring the difference in osteotomy between preoperative stimulated surgery and postoperative CT scan. The secondary goal of this trial is to assess the effect of adopting this system on improving the symmetry of the mandible, the postoperative pain score, the complication rate, and the satisfaction rating.

## Trial design {8}

**Fig. 1:**
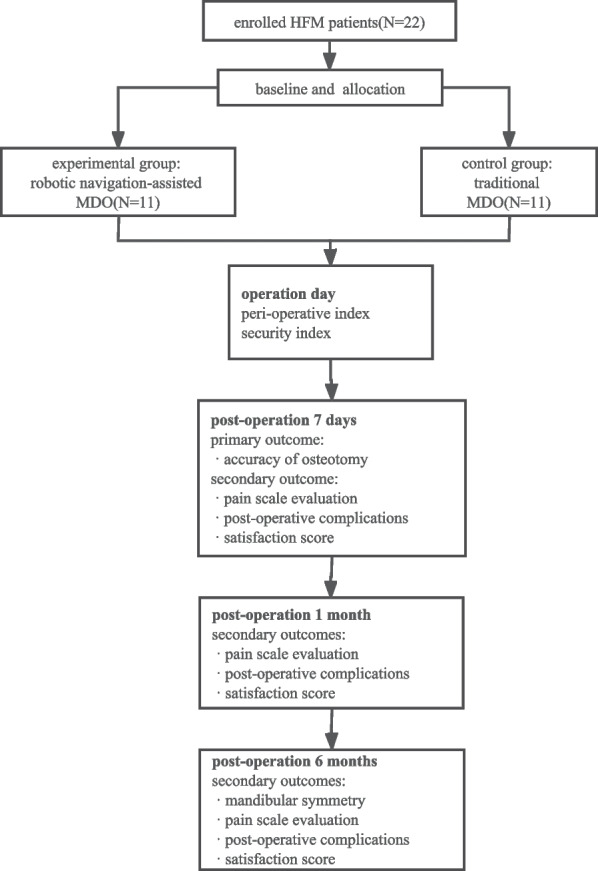
The trial framework. HFM, hemifacial microsomia; MDO, mandibular distraction osteogenesis

This trial is a prospective randomized controlled trial. There are two parallel groups, including the experimental group of patients receiving the EM navigation system based on the artificial intelligence for osteotomy and the control group having the MDO surgery in a traditional method that surgeons perform the osteotomy on their own experience. The allocation ratio of the two groups is 1:1. The framework of this trial is shown in Fig. 1.

## Methods: participants, interventions, and outcomes

### Study setting {9}

The candidate HFM patients enrolled in this trial are from Shanghai 9th People’s Hospital, School of Medicine, Shanghai Jiao Tong University. It is an academic hospital featured in plastic and reconstructive surgery. This trial has been registered in the Chinese Clinical Trial Registry Center (ChiCTR) with the number ChiCTR2200061565. (http://www.chictr.org.cn/edit.aspx?pid=168053&htm=4).

### Eligibility criteria {10}

The inclusion criteria include (1) the patients diagnosed with hemifacial microsomia and Pruzansky type II based on the 3D CT scan. In detail, type IIA refers to those whose ramus and condyle have abnormal morphology, but the condyle–glenoid fossa relationship is maintained; type IIB the ramus and condyle are hypoplastic and displaced toward the midline; (2) any genders between 3 and 18 years old; and (3) the informed consent obtained from parents/guardian.

The exclusion criteria include (1) younger than 3 years old; (2) diagnosed as Pruzansky type I (condyle and ramus are reduced in size, but the morphology is maintained) or type III (condyle and ramus are reduced in size, but the morphology is maintained) or other syndromes; (3) the informed consent was not signed; (4) combined with other congenital diseases; and (5) combined with contradictions of anesthesia.

The dropout criteria are as follows: (1) patient withdraws the consent; (2) failure to finish the whole treatment, including the examinations and the procedure; and (3) loss to follow-up.

### Who will take informed consent? {26a}

A specific investigator from the medical team will obtain written consent from the parents of the HFM patients who agree to participate.

### Additional consent provisions for collection and use of participant data and biological specimens {26b}

Not applicable, as no biological specimens would be collected.

### Interventions

#### Explanation for the choice of comparators {6b}

The patients of the control group received the mandibular distraction osteogenesis (MDO) surgery traditionally, in which the surgeon performs the osteotomy on their own experience based on preoperative design without navigation equipment. That is the most widely used method in the current operating theater.

#### Intervention description {11a}

**Fig. 2:**
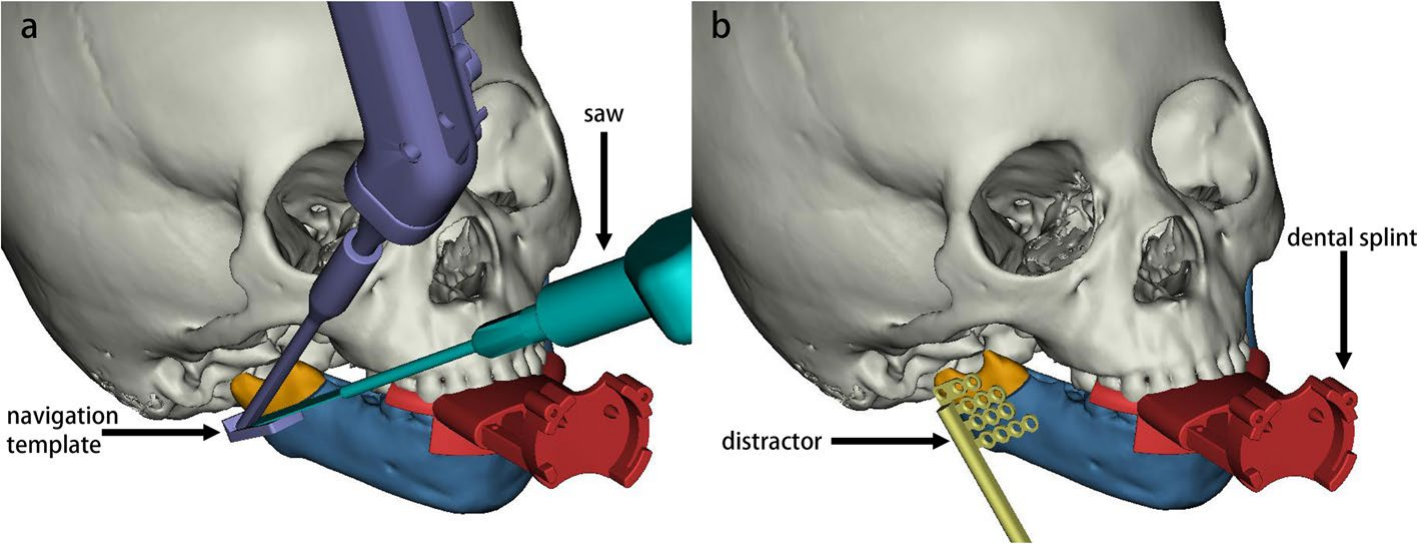
Preoperational surgical design is shown in Mimics (experiment group). **a** Design of osteotomy. **b** Design of distractor fixation. The position of the distractor is based on the osteotomy line. Orange: the proximal segment of the mandibular ramus. Blue: the distal segment of the mandible. Red: dental splint. Purple: robotic end with the template. Cyan: bone saw. Yellow: the distractor

After obtaining the consent form, all patients of each group receive the preoperative 3D CT scan, and the data will be stored in DICOM format and imported into the corresponding processing software (Mimics 21.0, Materialise, Belgium). The surgeons adopt the Mimics software to reconstruct the mandibular and design the osteotomy line to avoid the inferior alveolar nerve inside the mandibular as in the preoperative plan (Fig. 2).

For the control group, the surgeon locates the osteotomy line on the mandibular on their own experience based on the preoperative design image without the navigation, which is a traditional procedure of MDO for HFM patients.

**Fig. 3:**
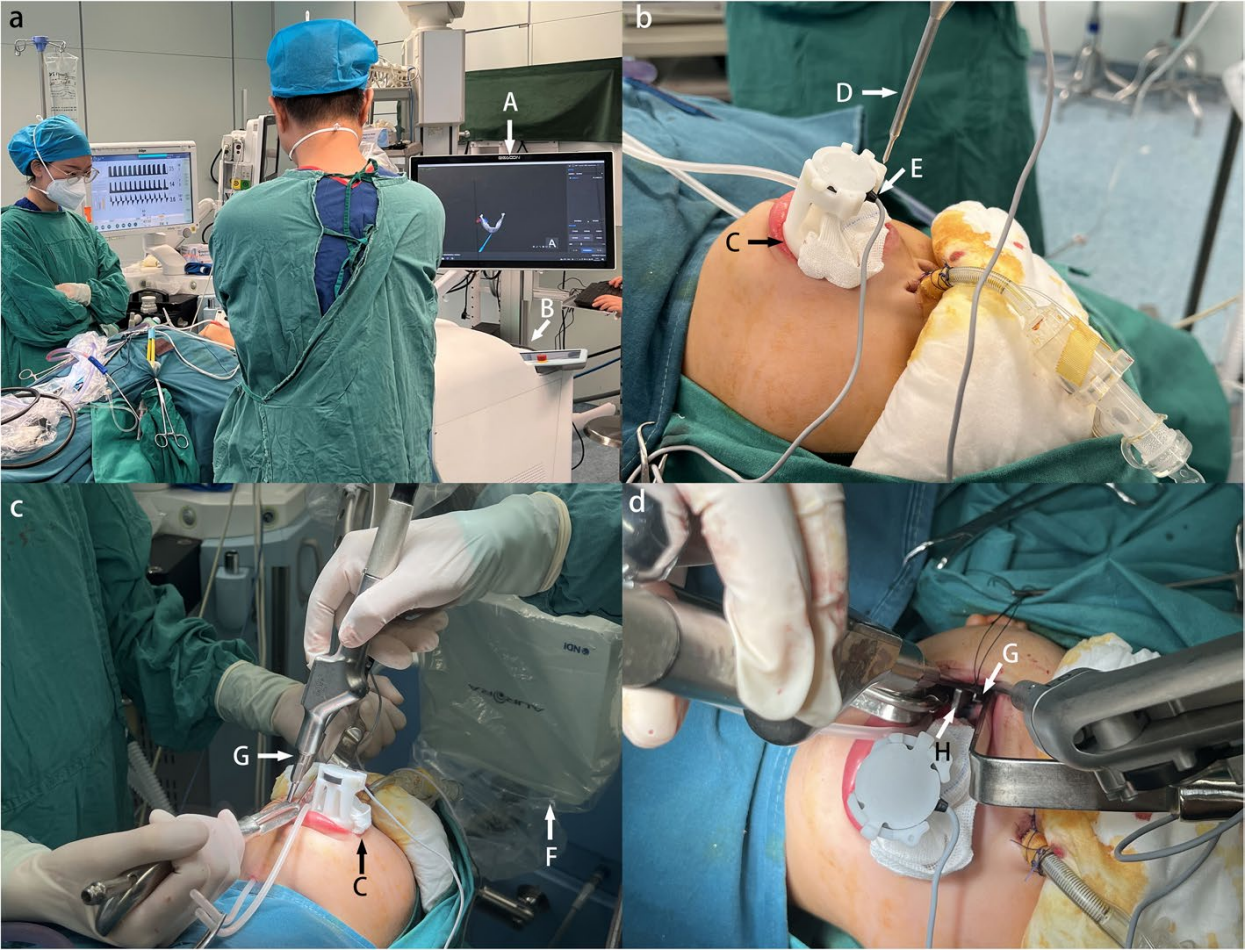
Robotic navigation-assisted MDO. **a** Surgical environment. **b** Verification of the system registration. The verification pinpointing at the registration point on the splint represents accurate registration. **c** Template in position. The surgeon is conducting osteotomy with the saw blade relying on the template. **d** Assisted osteotomy from the intraoral viewpoint. (A) Navigation system. (B) Computer base. (C) Dental splint. (D) Verification pin. (E) Electromagnetic receptor. (F) Electromagnetic generator. (G) Bone saw. (H) Template arriving at the designed osteotomy line

For the experiment group, the patients receive the preoperative CT scan equipped with a customized dental splint made based on the patient’s dental mold and assembled with four steel spheres with a diameter of 2 mm as the magnetic field tracking targets to navigation (Fig. 3a, b). Their preoperative plans generated by the Mimics software are transferred to the navigation system, which also stores the location information generated by the steel spheres of the dental splint. Besides, a 3D printed template is generated based on the designed osteotomy plan.

Before the osteotomy, the splint is mounted on the patient’s teeth as the registration piece is connected to the magnetic field of the navigation system. The 3D printed template will be clamped on the end of the robotic arm to locate precisely the osteotomy line according to the preoperative design documents stored and managed by the EM system. Then, the surgeon performs the osteotomy by moving the saw along the template (Fig. 3c, d).

The management of all patients is identical, and to reduce surgical bias, the chief surgeon in the control and experimental groups will be the same senior craniomaxillofacial specialist with more than 15 years of specialty practice and an average annual craniofacial surgery volume of 300 or more. There will be two attending surgeons assisting with the operation. The team training of the navigation-assisted surgery group is more than 100 h.

#### Criteria for discontinuing or modifying allocated interventions {11b}

The criteria for discontinuing or modifying include the malfunction of the EM system, surgical accident, and any situation that makes the surgeon need to finish the surgery as soon as possible. The patients’ demographic data that meet those criteria will still be collected.

#### Strategies to improve adherence to interventions {11c}

To improve retention, the participants will get a stipend if they return to the hospital for follow-up on time. In this study, children received surgical treatment with the participation of computer-assisted surgery, which reduced the surgical risks and complications. The distraction effect is evident in the early stage, which is conducive to the psychological development of school-age children. Some guardians worry that premature surgery is not conducive to nursing, and other psychological relief can also be alleviated. In addition, scientific research expenses are subsidized to reduce part of the economic burden.

#### Relevant concomitant care permitted or prohibited during the trial {11d}

There is no specific concomitant care or interventions prohibited during the trial. The two groups’ patients will receive standard perioperative care for their conditions.

#### Provisions for post-trial care {30}

After the trial, the surgical team will provide standard postoperative medical care for the participating patients.

### Outcomes {12}

#### Primary outcome

**Fig. 4:**
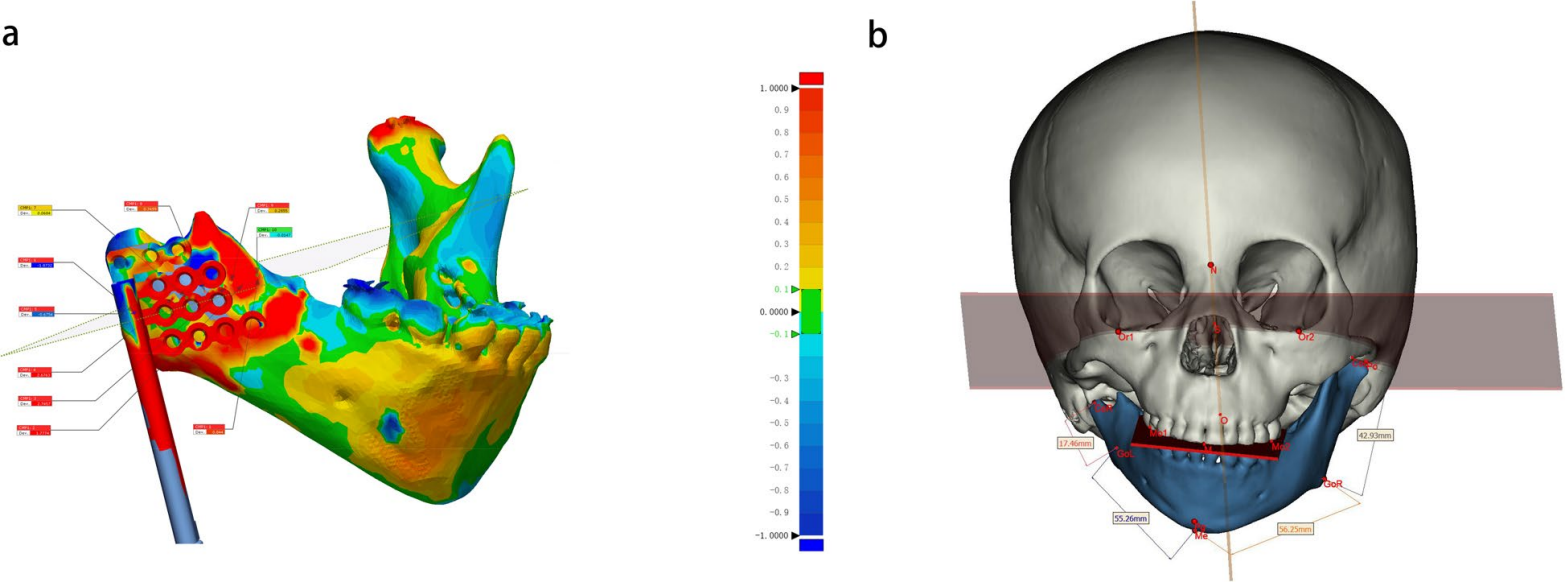
Postoperative imaging analysis. **a** Accuracy analysis by comparing the CT data 1 week after MDO with the preoperative design. **b** Symmetry analysis by comparing the image after removing the distractor with the original CT

The primary outcome will be the accuracy of osteotomy by comparing the preoperative design and postoperative CT. The two mental foremen and the condylion of the unaffected side are marked as the registration points. Altogether, 10 points on the osteotomy plane of each pre- and postoperative CT scan are selected to calculate the accuracy (Fig. 4a). Each patient’s data will be measured three times to avoid measurement bias, and the staff responsible for measurement are blinded to the patient’s grouping information.

#### Secondary outcomes

The secondary outcome includes the symmetry of the mandibular, pain scale, recording of surgical complications, and satisfactory score.

The symmetry of the mandibular is evaluated by three indicators: the angle of inclination, the mandibular ramus’s height, and the mandibular body’s length, which will be obtained from the CT 6 months after the surgery (Fig. 4b).

The angle of inclination: A horizontal plane (HP) is established by selecting bilateral suborbital points (Or) and the healthy superior external auditory canal points (Po). Establish the occlusal plane (OP) from the adjacent medial point of the maxillary central incisor to the medial buccal cusp of the bilateral first molar. Measure the angle between the occlusal plane and the horizontal plane, set as angle A. The smaller angle A indicates the smaller deviation angle of the mandible and maxilla, meaning the better symmetry of the lower face.

The height of mandibular ramus: The height of mandibular rami, that is, the distance between condylion (Co) and mandibular gonion (Go), is measured respectively. The distance of the affected side is set as *h*, and the distance of the healthy side is set as *H*. Comparing the values of the affected and the healthy sides. The smaller the difference, the better the bilateral symmetry.

The length of the mandibular body: The menton (me) is marked, and the length of the bilateral mandibular body, the distance from the bilateral gonion (Go) to the menton (Me), is measured respectively. The distance of the affected side is set as *l*, and the distance of the healthy side is set as *L*. Comparing the values of the affected and the healthy sides, the smaller the difference, the better the bilateral symmetry.

The pain scale: The evaluation time is 1 week, 1 month, and 6 months after surgery. The pain scale is an international visual analog scale, with a scale from 0 to 10 representing no pain to the most intense pain.

The postoperative complications during the trial will be recorded, including local infection, bone nonunion, scar hyperplasia, occlusal disorder, nerve injury, and tooth germ injury.

The satisfaction score was evaluated by a modified 5-point Likert table, with 1–5 indicating unsatisfactory to very satisfied. It will be recorded at the 1-week, 1-month, and 6-month follow-up.

Besides, the preoperative data are recorded, including surgical time, system installation time, hospital stay, blood loss volume, drainage volume, and drainage duration.

#### Security index

1. Incidence of organ and vascular injury events related to the system, including the following: (1) whether there is an intraoperative injury of adjacent organs caused by the failure of the system, including the injury of the masseter muscle, mouth, parotid gland, etc.; (2) whether there was massive bleeding caused by vascular injury caused by functional failure of the tested system during the operation. The incidence of injury events was defined as the proportion of subjects in the experimental control group who had an injury event to all subjects.
2. Incidence of adverse events during the trial.

An adverse event that occurs during the clinical trial, whether or not it is device-related, includes the following: (1) all suspected adverse device effects; (2) significant unrelated diseases, that is, certain symptoms or diseases that are not present at the time of entry into the study; (3) the worsening of an existing disease, that is, the simultaneous emergence of some new symptoms or signs. A pre-existing condition is defined as a condition or symptom that exists before the study begins and is initially recorded on the history/physical examination record prior to treatment; (4) accidental injury or accident; (5) any improper and unexpected event related to a medical device is an adverse device effect, that is, it is associated with the use of the study product. Any adverse event due to improper instructions for the use of a device, and any adverse event due to the user’s inappropriate operation, are included in this definition.

1. Incidence of device failure: (1) The console and machine communication suffers substantial interference. The movement of the manipulator fails and cannot be eliminated after reset and restart; (2) surgical clamps could not release the tissue; and (3) instrument installation alarm; intraoperative failure or damage of instruments.
2. Abnormal vital signs (body temperature, heart rate, respiration, mean arterial pressure (MAP)).

### Participant timeline {13}

See the participant timeline in Table 1.

**Table 1:** The participant and data collection timeline

**Time point**	Baseline and allocation (− day 7–0)	Operation day (day 1)	Postoperation		
			7 days	1 month	6 months
**Enrollment**					
Basic information	√				
Medical history	√				
Eligibility screen	√				
**Interventions**					
Traditional surgery		√			
Surgical robot		√			
**Primary outcome**					
Accuracy of osteotomy			√		
**Secondary outcome**					
The symmetry of mandibular					√
The pain scale evaluation			√	√	√
The postoperative complications			√	√	√
Satisfaction score			√	√	√
**Perioperative index**					
Surgical time		√			
System installation time		√			
Hospital stay			√		
Blood loss volume		√			
Drainage volume			√		
Drainage duration			√		
**Security index**					
Incidence of organ and vascular injury events related to the system		√			
Incidence of adverse events during the trial		√			
Incidence of device failure		√			
Abnormal vital signs		√			

### Sample size {14}

As this study is an exploratory clinical trial with the application of cutting-edge technology, a small sample randomized controlled method was designed to be carried out. Based on the literature review and our team’s preclinical experimental results, the sample size met the minimum requirements combined with the budget and actual situation of the project. The osteotomy accuracy in the previous study was 1.99 ± 1.32 mm and power *α* is set as 0.05 and (1 − *β*) as 0.9. Therefore, *n*1 = *n*2 = ${\left[\frac{{Z}_{\frac{\alpha }{2}}+{Z}_{\beta }}{\delta /\sigma }\right]}^{2}({Q}_{1}^{-1}+{Q}_{2}^{-1})$×½≈9.24 [13]. Considering the loss of follow-up (10%), the experimental and control groups will enroll 11 cases in each group.

### Recruitment {15}

The participants will be recruited at the Ninth People’s Hospital School of Medicine, Shanghai Jiao Tong University, one of the top plastic and reconstructive surgery centers nationally (the statement came from the 2022 China Hospital Specialty Comprehensive Ranking, a public interest discipline assessment project conducted by the Institute of Hospital Management of Fudan University as an independent third party), with hundreds of craniofacial surgeries performed yearly. When visiting our craniofacial clinic, HFM patients who meet the inclusion criteria will be introduced to this trial. If the patients or their parents are interested in attending the trial, they will be contacted by our staff to receive detailed information about this trial and sign the consent when they are hospitalized.

## Assignment of interventions: allocation

### Sequence generation {16a}

The SAS software is adopted for block randomization. It generates a random table for grouping, and the ratio of the experiment and control groups is 1:1. The statistician who does not participate in the operation divides the enrolled patients into groups according to the sequence of random numbers. The grouping information is sealed in the envelope and saved by the researchers who did not participate in the operation. All the enrolled subjects are treated in the experimental or control group according to the numbers shown in the random envelope to avoid bias caused by the non-random selection of samples.

### Concealment mechanism {16b}

The statistician in the surgery generates the random table for grouping by the SAS software. The researcher who does not participate in the surgery saves the grouping information in sealed envelopes. The random distribution list is confidential; every patient has a specific ID number. A specific staff is responsible for telling the surgeon which kind of operation the enrolled patient should receive. The grouping information and the identifiable information are saved in sealed envelopes separately.

### Implementation {16c}

First, the trial team’s investigators will be responsible for recruiting people who meet the inclusion criteria and obtaining permission. The study statisticians then do the randomized allocation and send the patient’s randomized number. Depending on the allocation, the surgeons will execute the procedure for the patient with or without the robotic navigation system. Finally, the engineer who is unaware of the allocation measures the outcomes.

## Assignment of interventions: blinding

### Who will be blinded {17a}

The trial will be conducted single blindly. Knowing which way to intervene, the operator is unable to be blinded. The recruited patients and outcome assessors have no idea if they are receiving surgery with the navigation system.

### Procedure for unblinding if needed {17b}

Only the patients and outcome assessors are blind in this single-blind trial; under no circumstances do they need to be unblinded.

## Data collection and management

### Plans for assessment and collection of outcomes {18a}

The image data from the preoperative and postoperative procedures will be transferred into Geomagic Control X (3D System, SC). The accuracy of the osteotomy plane is assessed after the registration.

The accuracy of the osteotomy between the preoperative plan and the postoperative result will be the primary outcome measure. The postoperative CT will be performed within 7 days following surgery, which is the most critical outcome for determining the efficacy of robotic navigation technology. Improvements in the symmetries of the mandibles will be assessed by comparing the CT 6 months after MDO and the preoperative ones with Mimics.

It will be organized for surgeons to record important information after surgery during the follow-up period for security measures such as intraoperative blood loss, operation time, hospitalization, possible blood transfusion, and complication rates.

### Plans to promote participant retention and complete follow-up {18b}

Children with hemifacial microsomia and their guardians are concerned about their prognosis since it affects function and appearance. Furthermore, collecting the patients’ contact information, such as their mobile phone number, WeChat ID, and email address, is common practice. Patients are also given our center’s phone number, which they may use to call our medical practitioner if they have any issues. We maintain constant contact with the patients in this manner to ensure proper follow-up. Also, certain patients must return to our facility for additional treatment of compounded issues, such as the occlusal condition. The medical team will give comprehensive pre- and postoperative care to encourage trial participants’ retention. All patients will be followed for 6 months for postoperative recovery and complications, and the PI will review the procedure and respond to the investigator’s report. The Clinical Research Center/Office of Quality Management of Shanghai 9th People’s Hospital, School of Medicine, Shanghai Jiao Tong University, will audit and verify the program’s data every quarter.

### Data management {19}

The image data will be imported into Geomagic Control X (3D System, SC) and Mimics (Materialise, Belgium). The accuracy of the osteotomy will be the primary outcome of the effectiveness of robotic technology. The precision of the osteotomy plane will be assessed by pre- and postoperative CT within 7 days following surgery. Mandibular symmetries will be assessed according to the CT 6 months after the surgery.

This team will provide for doctors to record essential information after surgery during the follow-up period for security indicators such as intraoperative blood loss, operation time, hospital time, any blood transfusion, and rates of complications.

### Confidentiality {27}

Every participant will be allocated an ID number, and their grouping data will be confidential and enveloped. All participants’ personal information will be collected by professional investigators and stored in secure cabinets with limited access. The digital data in the measurement program will be available exclusively via password to the team in charge of measurements.

### Plans for collection, laboratory evaluation, and storage of biological specimens for genetic or molecular analysis in this trial/future use {33}

Not applicable. No biological specimen is needed in this trial.

## Statistical methods

### Statistical methods for primary and secondary outcomes {20a}

The SAS software (version 9.4, SAS Institute, USA) will be used to analyze and process primary and secondary outcomes.* N* (%) and mean ± standard deviation (SD) with median and interquartile range shall describe all quantitative data. If the data follows a normal distribution, we use Student’s* t*-test to compare the two groups. For comparing enumeration data across groups, a sample size of less than 40 necessitates using Fisher’s exact test. *P* < 0.05 is deemed statistically significant.

### Interim analyses {21b}

Not applicable. Given how promptly the study could be conducted, there is little risk of significant complications arising.

### Methods for additional analyses (e.g., subgroup analyses) {20b}

Not applicable. No subgroup analysis will be performed.

### Methods in analysis to handle protocol non-adherence and any statistical methods to handle missing data {20c}

The analysis in this trial will be based on the intention-to-treat (ITT) principle. The ITT will also handle those who meet the withdrawal criteria, including demographic information. If that data is lacking, the statistician will use the last observation carried forward (LOCF) method to finish the analysis.

### Plans to give access to the full protocol, participant-level data, and statistical code {31c}

In order to view the entire protocol, please visit https://www.chictr.org.cn/showproj.aspx?proj=38142. After the study results are published, the relevant data and statistical code will be published.

## Oversight and monitoring

### Composition of the coordinating center and trial steering committee {5d}

This is a single-center trial. Skilled investigators on the trial staff handle enrollment, clinical data gathering, digital data measurement, and the surgical team performing the procedure and providing medical care. The trial’s PI will check in with the investigators monthly to hear their progress report and ensure everything is running well. The data and research progress will be reviewed and checked every quarter by the trial steering committee from the Clinical Research Center/Office of Quality Management at Shanghai 9th People’s Hospital, School of Medicine, Shanghai Jiao Tong University.

### Composition of the data monitoring committee, its role, and reporting structure {21a}

Staff and specialists from the Clinical Research Center/Office of Quality Management at Shanghai 9th People’s Hospital, School of Medicine, Shanghai Jiao Tong University, will conduct quarterly audits and reviews of the study data as part of the trial’s data monitoring committee. There are no competing interests.

### Adverse event reporting and harms {22}

The available literature on robotic navigation has documented no adverse outcomes in other surgical procedures. Suppose there are any medical issues in either group throughout the follow-up period. In that case, the team’s clinical doctor will document and treat them before reporting to the ethics committee a week later.

### Frequency and plans for auditing trial conduct {23}

PI will review the procedure and hear the results of this test every month. Data audit and verification of the program will be conducted quarterly by the Clinical Research Center/Office of Quality Management at Shanghai 9th People’s Hospital, School of Medicine, Shanghai Jiao Tong University.

### Plans for communicating important protocol amendments to relevant parties (e.g., trial participants, ethical committees) {25}

The protocol’s revisions will be submitted to the ethics committee for review and approval before implementation.

## Dissemination plans {31a}

Patients’ confidentiality and interests will be safeguarded while the study’s results are shared through scientific publications and conference presentations. Anything that could compromise the blind status of the trial will not be made public until after it has concluded. Dr. Gang Chai, the PI, will take charge of the publication process and receive credit for his work.

## Discussion

This article describes a randomized controlled experiment that intends to investigate the efficacy of EM robotic navigation for treating HFM. Although MDO is complex, osteotomy requires increased precision and stability to produce desirable functional and attractive results [5]. With the help of a robotic navigation system based on artificial intelligence, surgeons can now have real-time navigation based on a 3D model, combining the best of both worlds. Evidence found that it improves depth perception and hand–eye coordination in various surgical procedures. The trial’s results could pave the way for more widespread use of EM robotic navigation technology in MDO procedures. This research’s sample size is relatively tiny compared to past experimental studies on the use of robotic technology in various types of surgeries; hence, the next step would be to conduct a larger sample size and multi-center trial. Data from this study will be used to refine the trial design necessary to demonstrate navigation’s therapeutic utility and to sustain advances made in MDO for HFM.

## Trial status

Protocol version = 1.1

Date of register = December 16, 2021

Date recruitment began = December 16, 2021.

Estimate completion date = December 2024.

## Data Availability

The primary investigators have access to the final trial dataset, including the PI, the attending surgeons, the statisticians, and the outcome accessors. The complete data will be made available when this trial is complete and published. Application for the data to be released should be made in contact with GC (PI).

## Abbreviations

HFM: Hemifacial microsomia
TMJ: Temporomandibular joint
MDO: Mandibular distraction osteogenesis
CAD: Computer-aided design
EM: Electromagnetic
MSRS: Maxillofacial surgical robot system
CT: Computed tomography
3D: Three-dimensional
DICOM: Digital Imaging and Communication in Medicine
HP: Horizontal plane
ITT: Intention-to-treat
LOCF: Last observation carried forward
PI: Principal investigator
